# Identification and molecular mechanism of palmitoylation-related biomarkers in obstructive sleep apnea

**DOI:** 10.3389/fneur.2025.1499573

**Published:** 2025-08-29

**Authors:** Yiguang Hong, Suyi Zeng, Xueqian Wang, Wei Kang, Bihua Chen, De Lan, Xuemei Wei

**Affiliations:** Department of Otolaryngology-Head and Neck Surgery, Clinical Medical College & Affiliated Hospital of Chengdu University, Chengdu University, Chengdu, China

**Keywords:** obstructive sleep apnea, palmitoylation, nomogram, HIF1A, PDIA3

## Abstract

**Introduction:**

Palmitoylation influences patients with obstructive sleep apnea (OSA) by modulating amyloid-β production. However, the involvement of palmitoylation-related genes (PRGs) in OSA remains unclear. This study aims to investigate this mechanism using bioinformatics approaches.

**Methods:**

Datasets GSE38792 and GSE135917 were retrieved from the Gene Expression Omnibus (GEO) database. Differentially expressed PRGs (DE-PRGs) were identified through differential expression analysis and weighted gene co-expression network analysis (WGCNA). Candidate genes were pinpointed using the max cluster centrality method in cytoHubba. Biomarkers were selected through machine learning algorithms, expression profiling, and ROC analysis, with diagnostic potential evaluated using a nomogram. Further insights into the role of biomarkers in OSA were provided through enrichment analysis, molecular regulatory network construction, and drug prediction.

**Results:**

HIF1A and PDIA3 emerged as potential biomarkers, with the nomogram showing high predictive accuracy for OSA. Enrichment analysis revealed that HIF1A and PDIA3 were co-enriched in pathways such as “focal adhesion,” “olfactory transduction,” “RNA degradation,” “spliceosome,” and “ubiquitin-mediated proteolysis.” A lncRNA-miRNA-mRNA regulatory network was constructed, featuring multiple regulatory pairs, including CYTOR-hsa-miR-1-3p-HIF1A and CYTOR-hsa-miR-1-3p-PDIA3. Drug prediction analysis identified potential compounds targeting HIF1A, such as klugine, puupehenone, and isocephaeline.

**Conclusion:**

HIF1A and PDIA3 were highlighted as significant potential biomarkers, providing valuable insights into the molecular mechanisms of palmitoylation in OSA and potential therapeutic targets.

## 1 Introduction

Obstructive sleep apnea (OSA) is characterized by apnea and hypoventilation caused by the collapse and obstruction of the upper airway during sleep, often accompanied by symptoms such as snoring, disrupted sleep architecture, frequent occurrence of oxygen desaturation, and daytime sleepiness. Affecting nearly 1 billion individuals worldwide, OSA represents a significant global public health issue (1, 70). The condition leads to intermittent hypoxia (IH), hypercapnia, sleep fragmentation, and reduced oxygen saturation during sleep (2), substantially increasing the risk of central nervous system dysfunction, including depression, anxiety, and memory deficits (3). Furthermore, it elevates the risk of cardiovascular diseases such as hypertension and abnormal heart rate variability (4). OSA also increasingly affects younger populations (5). Moreover, IH and fragmented sleep in OSA contribute to the development of cancer and type 2 diabetes, as well as metabolic disorders such as obesity, insulin resistance, and dyslipidemia (1, 2). Recurrent upper airway obstruction in OSA can exacerbate discomfort due to prolonged IH (6). Despite its high prevalence, treatment options for OSA remain limited. Currently, positive airway pressure remains the first-line therapy for moderate to severe cases, with continuous positive airway pressure being the most widely used. However, its tolerance is generally poor, leading to relatively low patient adherence. Other treatments, such as weight loss, positional therapy, exercise, dietary adjustments, surgery, oral appliances, and upper airway stimulation, also present significant limitations (7). Currently, therapeutic drugs, including antihypertensives, antidiabetic agents, anti-inflammatory drugs, immunosuppressants, antidepressants, and synthetic cannabinoids, are used, but no drug has proven efficacy for OSA treatment (8). Consequently, advancing the understanding of the molecular mechanisms underlying OSA and identifying potential biomarkers are crucial for improving clinical treatment outcomes.

Protein palmitoylation is a reversible post-translational modification catalyzed by palmitoyltransferases and depalmitoyltransferases, playing a pivotal role in regulating protein localization, stability, and function (9). This modification is mediated by the zinc finger protein DHHC (ZDHHC) family, consisting of 23 distinct proteins in mammals that catalyze the reversible attachment of palmitate (10). In contrast, depalmitoylation is primarily driven by three distinct families, encompassing seven genes (9, 11). Palmitoylation involves the formation of thioester bonds between the palmitate moiety and the sulfhydryl group of cysteine residues, affecting over 4,000 human proteins (11). As a reversible modification, protein palmitoylation participates in various biological processes related to multiple diseases, including cancer, diabetes, Alzheimer’s disease, and inflammation (9, 10, 12, 13). The hypopnea index in OSA is associated with 65 proteins, and analysis of 254 serum proteins from patients with OSA revealed a prominent insulin-associated protein signature, alongside elevated insulin levels in their blood (15). Notably, several components involved in insulin secretion and action have been linked to palmitoylation (12). Additionally, amyloid-β expression in the cerebrospinal fluid of patients with OSA has been found to be increased (15, 16), with palmitoylation playing a role in amyloid-β production (17). These findings suggest that palmitoylation may significantly contribute to the development of OSA, but the precise biological mechanism underlying its role remains unclear.

This study utilized transcriptome sequencing data from public databases and applied bioinformatics approaches to analyze and identify palmitoylation-related genes (PRGs) in OSA. Potential biomarkers were explored, and their biological functions were investigated, providing new insights for early prevention and the development of clinical therapeutic strategies for patients with OSA.

## 2 Materials and Methods

### 2.1 Data extraction

The OSA-related datasets, GSE38792 and GSE135917, were retrieved from the Gene Expression Omnibus (GEO) database,^1^ with a sequencing platform of GPL6244. The GSE38792 dataset (training set) included visceral adipose tissue samples from 10 patients with OSA and 8 healthy controls. A total of 66 samples were included in the GSE135917 dataset (validation set). After excluding 48 treated samples, 10 untreated OSA samples and 8 normal control samples were retained for analysis. Detailed sample information of the two datasets is shown in Supplementary Table 1. Additionally, 30 PRGs were sourced from the literature (9, 18), comprising 23 palmitoyl acetyltransferase genes and 7 de-palmitoyl acetyltransferase genes.

### 2.2 Differential expression analysis

Differential expression analysis of the GSE38792 dataset was performed using the limma package (v 3.54.0) (19) to identify differentially expressed genes (DEGs) between OSA and normal groups, with criteria set at |log_2_foldchange (FC)| >0.5 and *p* < 0.05. Visualization of DEGs was achieved using the ggplot2 package (v 3.4.1) (20) to generate a volcano plot. To further visualize the DEG trends, a heat map was created with the ComplexHeatmap package (v 2.15.1) (21), highlighting the top 10 upregulated and downregulated genes.

### 2.3 Weighted gene co-expression network analysis

The single-sample gene set enrichment analysis (ssGSEA) algorithm from the GSVA package (v 1.42.0) (22) was applied to calculate PRG scores for the OSA and normal samples in the GSE38792 dataset. A Wilcoxon test was then conducted to assess the differences in PRG scores between OSA and normal samples (*p* < 0.05).

Next, weighted gene co-expression network analysis (WGCNA) was performed using the WGCNA package (v 1.70-3) (23) to identify key gene modules associated with PRGs. Initially, the GSE38792 dataset was subjected to cluster analysis using the GoodSamplesGenes function to detect potential outlier samples. Outliers were excluded to ensure the accuracy and reliability of the subsequent analyses. The optimal soft threshold (β) was determined to ensure the gene interactions conformed to a scale-free network distribution, with a scale-free fit index (*R*^2^) of 0.85 and mean connectivity approaching 0. The dynamic tree-cutting algorithm was then applied, setting the minimum number of genes per module to 100 and mergeCutHeight to 0.4 (55% similarity), resulting in the clustering of genes into distinct modules. PRG scores were treated as phenotypic traits, and Pearson correlation analyses between gene modules and phenotypic traits were performed using the psych package (v 2.2.9) (24) with thresholds set at |*r*| > 0.4 and *p* < 0.05. Modules showing the highest positive and negative correlations with PRG scores were selected as key modules. The key module genes were identified by screening for |module membership (MM)| >0.8 and |gene significance (GS)| >0.2.

### 2.4 Identification of candidate genes

Differentially expressed PRGs (DE-PRGs) were identified by intersecting key module genes with DEGs using the VennDiagram package (v 1.7.1) (25). To explore the biological functions and signaling pathways associated with DE-PRGs, Gene Ontology (GO) and Kyoto Encyclopedia of Genes and Genomes (KEGG) enrichment analyses were performed using the clusterProfiler package (v 4.2.2) (26), with significance set at *p* < 0.05. The GO system was divided into three categories: biological process (BP), molecular function (MF), and cellular component (CC). The enrichment results were visualized using the enrichplot package (v 1.18.3) (27). To better understand the protein-level interactions among DE-PRGs, a protein–protein interaction (PPI) network was constructed using the Search Tool for the Retrieval of Interacting Genes (STRING) database^2^ with a confidence score ≥ 0.4. The PPI network results were then imported into Cytoscape software (v 3.5.2) (28), and the maximum cluster centrality (MCC) algorithm in the cytoHubba plug-in was applied. The top 10 genes from the MCC algorithm were selected as candidate genes for further analysis.

### 2.5 Recognition and localization of biomarkers

Candidate genes were further analyzed using the least absolute shrinkage and selection operator (LASSO) regression, implemented with the glmnet package (v 4.1-2) (29). The optimal *λ* value was determined through 10-fold cross-validation to identify the most relevant feature genes. Simultaneously, the Support Vector Machine-Recursive Feature Elimination (SVM-RFE) algorithm, implemented with the e1071 package (v 1.7.13) (30), was used for additional gene selection. This approach ranked genes based on their significance and evaluated the error rate and accuracy for different gene combinations in each iteration. The optimal combination of genes was selected based on the lowest error rate, and the corresponding genes were identified as feature genes. Candidate biomarkers were determined by overlapping the results from LASSO and SVM-RFE analyses. These biomarkers were then validated through expression analysis and receiver operating characteristic (ROC) curve assessment. ROC analysis was performed using the pROC package (v 1.18.0) (31). The ability of the candidate biomarkers to differentiate OSA samples from normal controls was evaluated based on the area under the curve (AUC) value in the ROC curve, with an AUC value >0.7 considered indicative of substantial predictive performance. Biomarkers were defined as such if they met the following criteria: (i) consistent expression trends in both the GSE38792 and GSE135917 datasets, (ii) significant expression differences between OSA and control groups in both datasets (*p* < 0.05), and (iii) AUC values exceeding 0.7 in both datasets. Finally, the chromosomal localization of the candidate biomarkers was visualized using the RCircos package (v 1.2.2) (32).

### 2.6 Construction and validation of nomogram

The diagnostic potential of biomarkers for OSA was further assessed by constructing a nomogram as a diagnostic model using the rms package (v 6.7.1) (33) within the entire GSE38792 dataset. A calibration curve was plotted to evaluate the predictive performance of the nomogram, with better predictive accuracy indicated by closer alignment to the diagonal. A mean absolute error (MAE) of less than 0.1 indicated minimal discrepancy between actual and predicted disease risks, demonstrating high accuracy of the nomogram model in predicting OSA. Additionally, ROC curves, decision curves, and clinical impact curves (CIC) were created to further assess the nomogram’s predictive capability. Decision curves were generated using the ggDCA package (v 1.2).^3^

### 2.7 Functional enrichment analysis

Genes related to the function of the biomarkers were predicted using the GeneMANIA database.^4^ To explore the biological functions of the biomarkers potentially involved in OSA pathogenesis, patients in the GSE38792 dataset were stratified into high and low expression groups based on the median expression level of each biomarker. Differential expression analysis was then conducted using the limma package. Genes were ranked in descending order according to their logFC values. GSEA was performed for each biomarker using the “c2.cp.kegg.v7.5.0.symbols” gene set from the Molecular Signatures Database (MSigDB) as a reference, with thresholds set at a false discovery rate (FDR) <0.25, *p* < 0.05, and |normalized enrichment scores (NES)| >1.

### 2.8 Establishment of molecular regulatory networks and drug prediction

To further investigate the complex regulatory mechanisms underlying biomarker expression, the miRNet database^5^ was utilized to predict microRNAs (miRNAs) targeting the biomarkers. Core miRNAs were selected based on their targeting of multiple biomarkers, and their upstream long non-coding RNAs (lncRNAs) were subsequently predicted using the miRNet database. Cytoscape software was employed to construct lncRNA-miRNA-mRNA regulatory networks, retaining nodes with a degree ≥3. Additionally, drugs targeting these biomarkers were identified through the drug-gene interaction database (DGIdb)^6^. Based on the interaction scores between biomarkers and drugs, the top 20 drugs were selected, and a biomarker-drug network was created.

### 2.9 Statistical analysis

**Figure 1:**
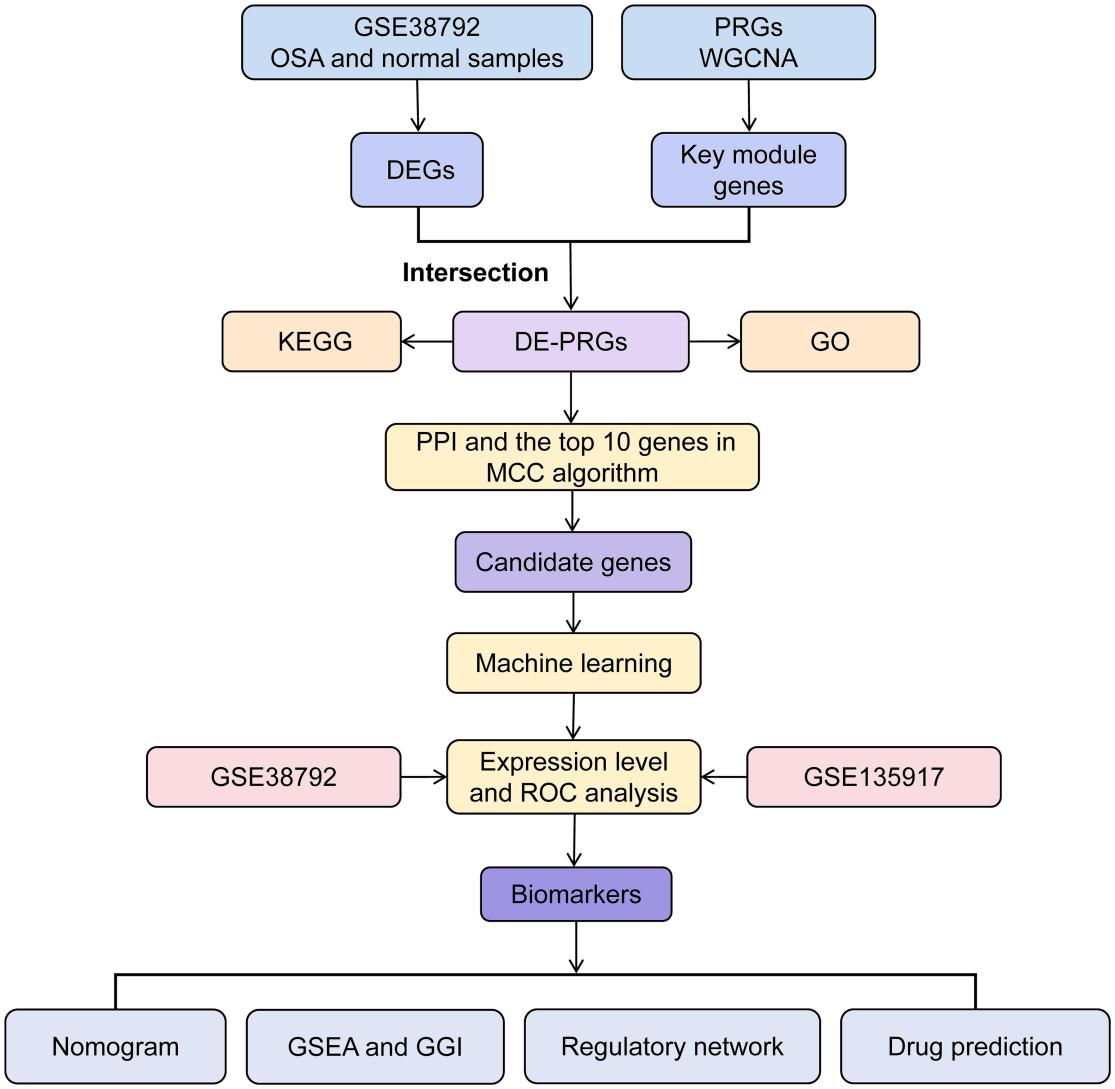
The analysis process of this study.

Data processing and analysis were conducted using R software (v 4.1.0). Differences between groups were analyzed using the Wilcoxon test, with a *p*-value <0.05 considered statistically significant. The overall analysis process of this study was shown in Figure 1.

## 3 Results

### 3.1 Identification of 367 DEGs and 2,303 key module genes

**Figure 2:**
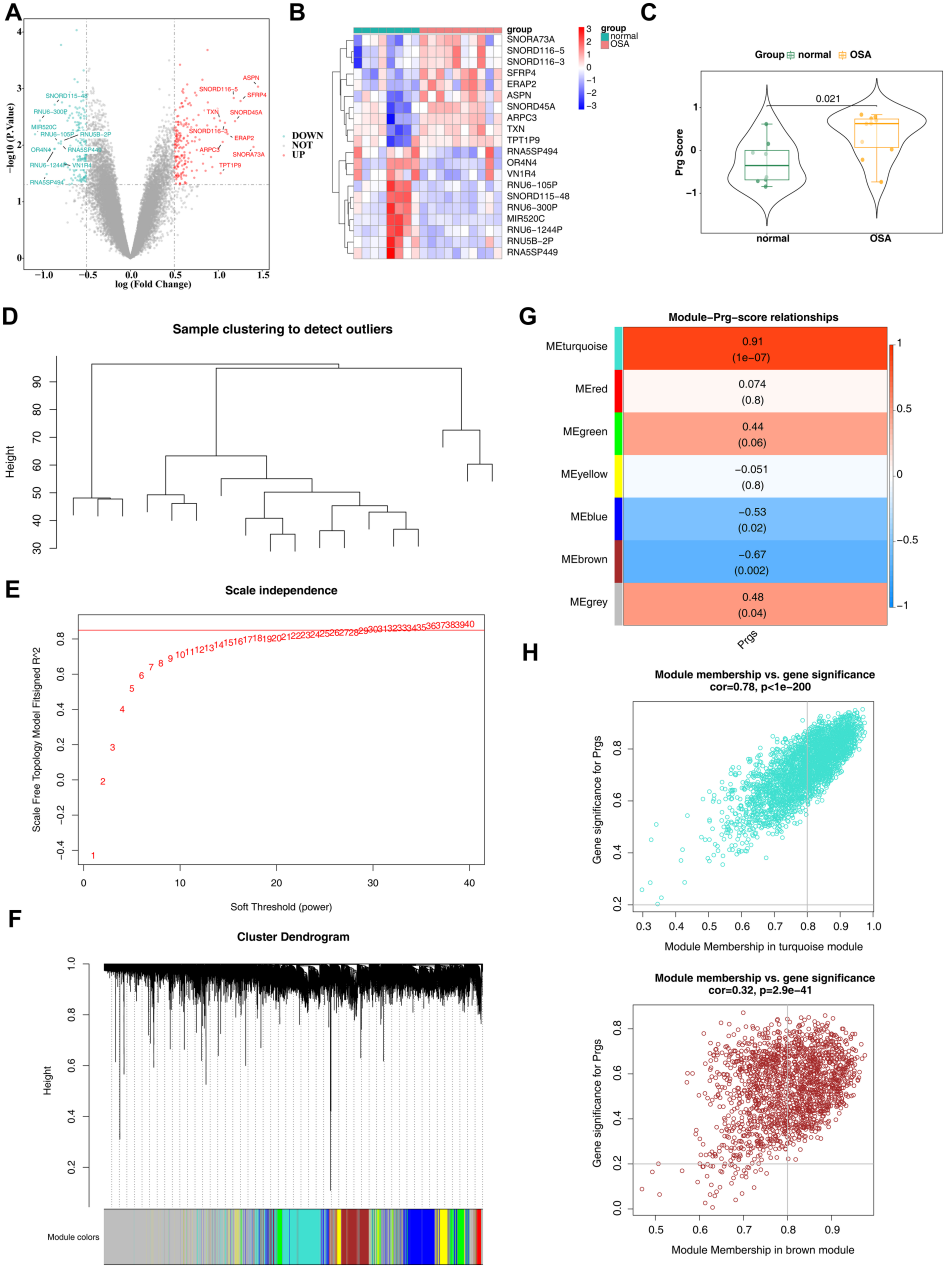
Identification of differentially expressed genes in OSA. **(A)** Volcano plot showing differentially expressed genes, with red indicating upregulated genes and blue indicating downregulated genes. The top 10 genes with the largest differences are labeled. **(B)** Heatmap displaying the differential expression of genes. **(C)** Differences in PRG scores between OSA and normal groups. **(D)** Cluster analysis of all samples in the training set, GSE38792. **(E)** Determination of the optimal soft threshold in WGCNA analysis. **(F)** Identification of six gene modules through dynamic tree cutting. **(G)** Heatmap of the correlation between gene modules and PRG scores. **(H)** Significance analysis of MEturquoise and MEbrown module members and their associated genes.

In the GSE38792 dataset, 367 DEGs were identified between the OSA and normal groups, consisting of 187 up-regulated genes and 180 down-regulated genes (|log_2_FC| >0.5 and *p* < 0.05) (Figures 2A,B). The Wilcoxon test revealed a significant difference in PRG scores between OSA and normal groups, with notably higher scores in the OSA group (*p* = 0.01) (Figure 2C), suggesting a link between palmitoylation and the development of OSA. WGCNA was performed to identify key module genes associated with PRGs. Clustering results showed no outlier samples in the GSE38792 dataset, enabling the continuation of analysis (Figure 2D). Using a soft threshold (β) of 30, determined based on *R*^2^ = 0.85 and connectivity approaching 0 (Figure 2E), six gene modules were identified through the dynamic tree cutting algorithm (Figure 2F). Further analysis identified the turquoise module [module eigengene (ME) turquoise; *r* = 0.91, *p* < 0.001] and brown module (ME brown; *r* = −0.67, *p* = 0.002) as key modules due to their strong correlations with PRG scores (Figure 2G). A total of 2,303 key module genes were filtered (Figure 2H).

### 3.2 A total of 10 candidate genes were identified

**Figure 3:**
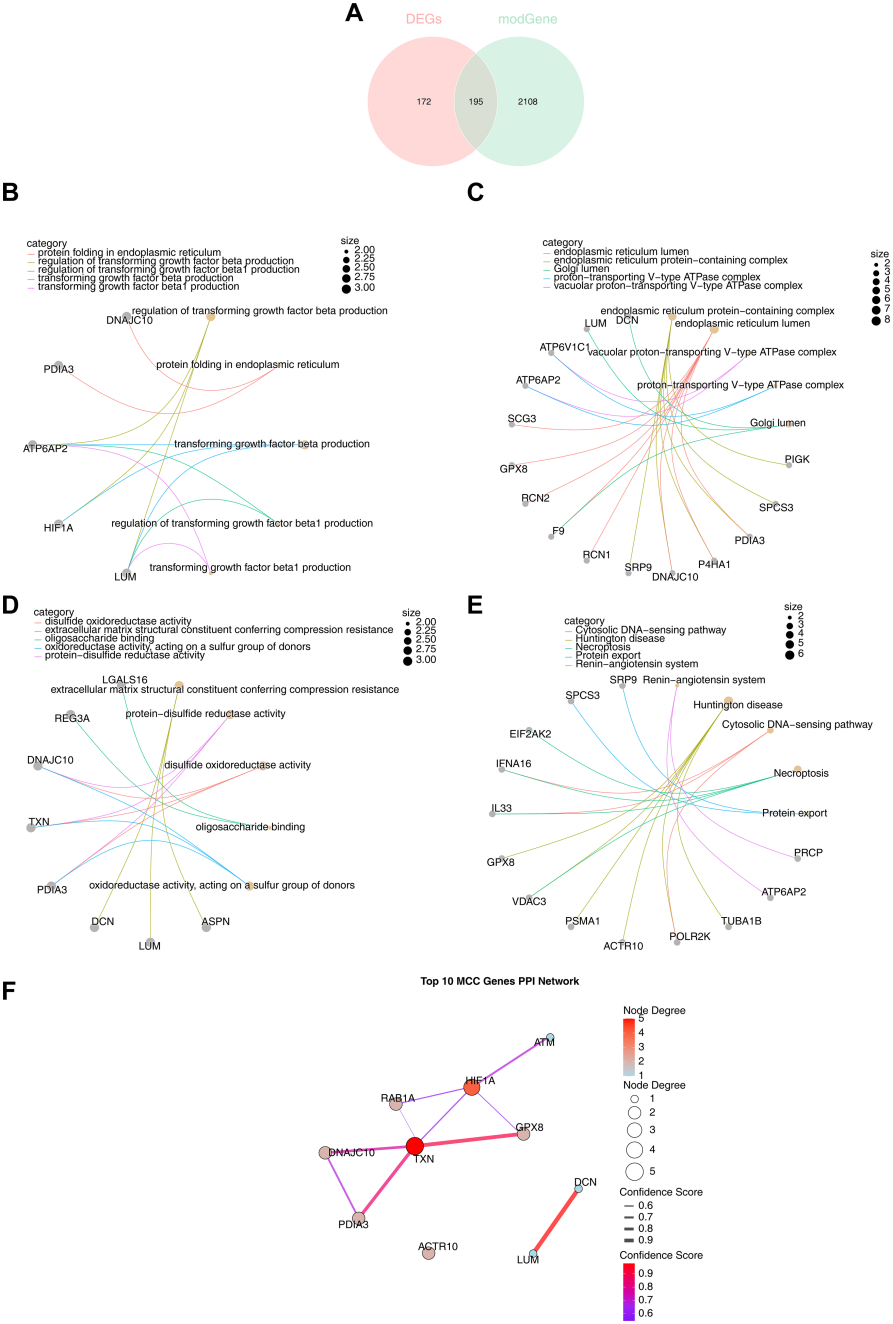
Functional enrichment analysis of DE-PRGs. **(A)** A total of 195 DE-PRGs were obtained by intersecting 2,303 key module genes and 367 DEGs. **(B)** GO enrichment analysis: biological processes (BP) involving 95 pathways. **(C)** GO enrichment analysis: cellular components (CC) associated with 21 pathways. **(D)** GO enrichment analysis: molecular functions (MF) linked to 19 pathways. **(E)** KEGG enrichment analysis revealing associated pathways. **(F)** Top 10 candidate genes identified by the maximum cluster centrality (MCC) algorithm from the PPI network.

By intersecting the 2,303 key module genes with the 367 DEGs, 195 DE-PRGs were identified (Figure 3A). These DE-PRGs were significantly enriched in 135 GO biological functions (95 BPs, 19 MFs, and 21 CCs) and 12 KEGG pathways. In GO-BP, the DE-PRGs were notably enriched in functions such as “regulation of transforming growth factor beta production,” “regulation of transforming growth factor beta1 production,” and “protein folding in the endoplasmic reticulum” (Figure 3B). In GO-CC, DE-PRGs were significantly involved in cellular processes like “endoplasmic reticulum lumen,” “proton-transporting V-type ATPase complex,” and “Golgi lumen” (Figure 3C). In GO-MF, DE-PRGs contributed to critical molecular functions including “disulfide oxidoreductase activity,” “protein-disulfide reductase activity,” and “oligosaccharide binding” (Figure 3D). KEGG pathway enrichment revealed significant involvement in pathways such as the “cytosolic DNA-sensing pathway,” “necroptosis,” and “protein export” (*p* < 0.05) (Figure 3E).

A PPI network was constructed, comprising 102 nodes and 57 edges, with multiple genes interacting, including *GPX8*, *HIF1A*, *ERO1A*, and *ACTB* (Supplementary Figure 1). The MCC algorithm identified 10 candidate genes: *PDIA3*, *RAB1A*, *TXN*, *DCN*, *HIF1A*, *ACTR10*, LUM, *DNAJC10*, *ATM*, and *GPX8* (Figure 3F).

### 3.3 HIF1A and PDIA3 were identified as biomarkers

**Figure 4:**
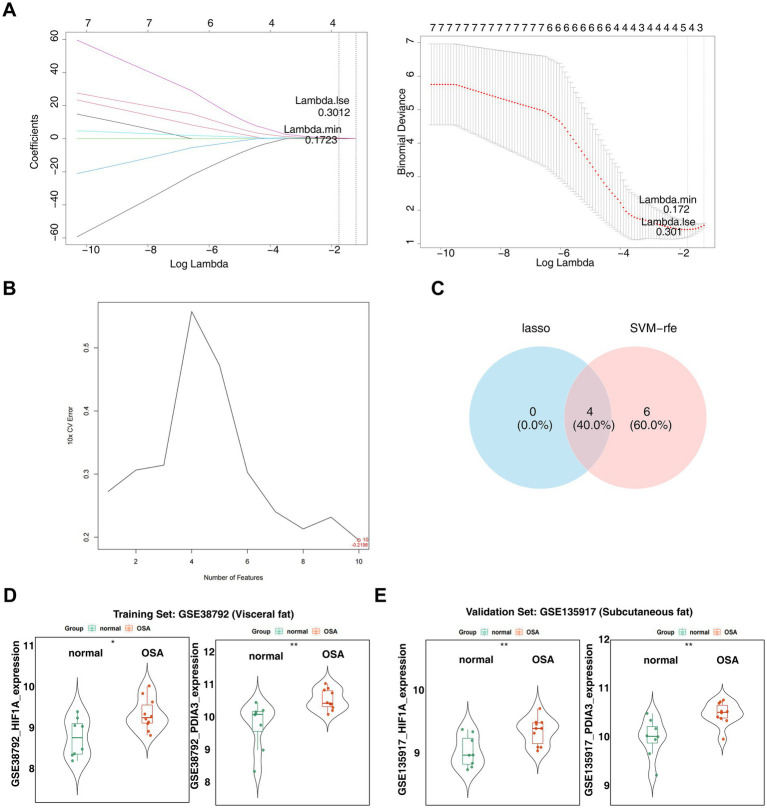
Identification of biomarkers. **(A)** The left side displays the LASSO coefficient plot,with the horizontal axis represents the logarithm of the lambda values and the vertical axis shows the variable coefficients. The right side presents the cross-validation results of the LASSO regression analysis, with the horizontal axis indicating log (lambda) and the vertical axis displaying model error. **(B)** SVM-RFE analysis for the 10 genes, with horizontal axis indicating the number of genes and vertical axis representing the error value. **(C)** Venn diagram showing the intersection of feature genes obtained from LASSO regression and SVM-RFE analyses. **(D)** Expression analysis of candidate biomarkers in the GSE38792 dataset. ^*^p < 0.05 and ^**^p < 0.01. **(E)** Expression analysis of candidate biomarkers in the GSE135917 dataset. ^*^p < 0.05 and ^**^p < 0.01.

**Figure 5:**
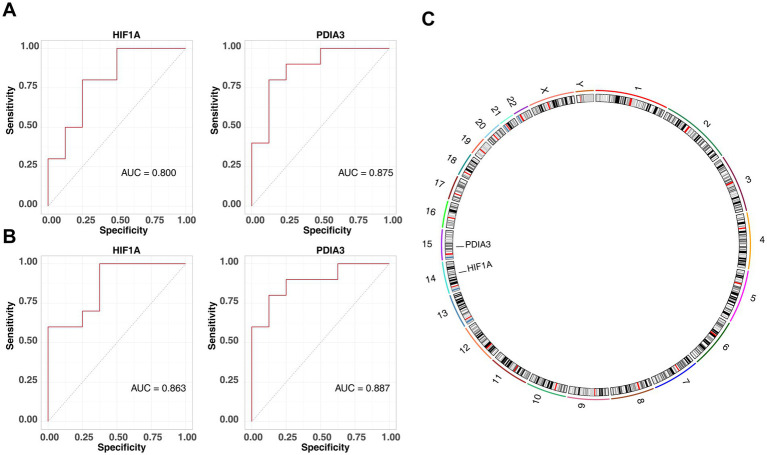
Identification of biomarkers. **(A)** ROC curve for HIF1A and PDIA3 in the GSE38792 dataset, with AUC >0.8. **(B)** ROC curve for HIF1A and PDIA3 in the GSE135917 dataset, with AUC >0.8. **(C)** Chromosomal localization analysis of HIF1A and PDIA3.

When the lambda.min value in the LASSO algorithm was set at 0.1723, four feature genes—*HIF1A*, *LUM*, *PDIA3*, and *TXN*—retained non-zero regression coefficients (Figure 4A). Additionally, the SVM-RFE algorithm identified *PDIA3*, *RAB1A*, *TXN*, *DCN*, *HIF1A*, *ACTR10*, *LUM*, *DNAJC10*, *ATM*, and *GPX8* (Figure 4B). By overlapping the two sets of feature genes, four candidate biomarkers (*HIF1A*, LUM, *PDIA3*, and *TXN*) were selected (Figure 4C). Expression analysis confirmed consistent upregulation of *HIF1A* and *PDIA3* in the OSA groups from both the GSE38792 (Figure 4D) and GSE135917 datasets (*p* < 0.05) (Figure 4E). In both datasets, AUC values for *HIF1A* and *PDIA3* surpassed 0.8 (Figures 5A,B). However, LUM and TXN showed no significant differential expression between the OSA group and the control group in the GSE135917 dataset (*p* > 0.05), with their AUC values both below 0.7 (LUM: AUC = 0.650; TXN: AUC = 0.625) (Supplementary Figure 2). Therefore, LUM and TXN were excluded, and HIF1A and PDIA3 were selected as biomarkers. Chromosomal localization analysis revealed that *HIF1A* is located on chromosome 14, while *PDIA3* is on chromosome 15 (Figure 5C).

### 3.4 A nomogram with excellent predictive ability for OSA was created

**Figure 6:**
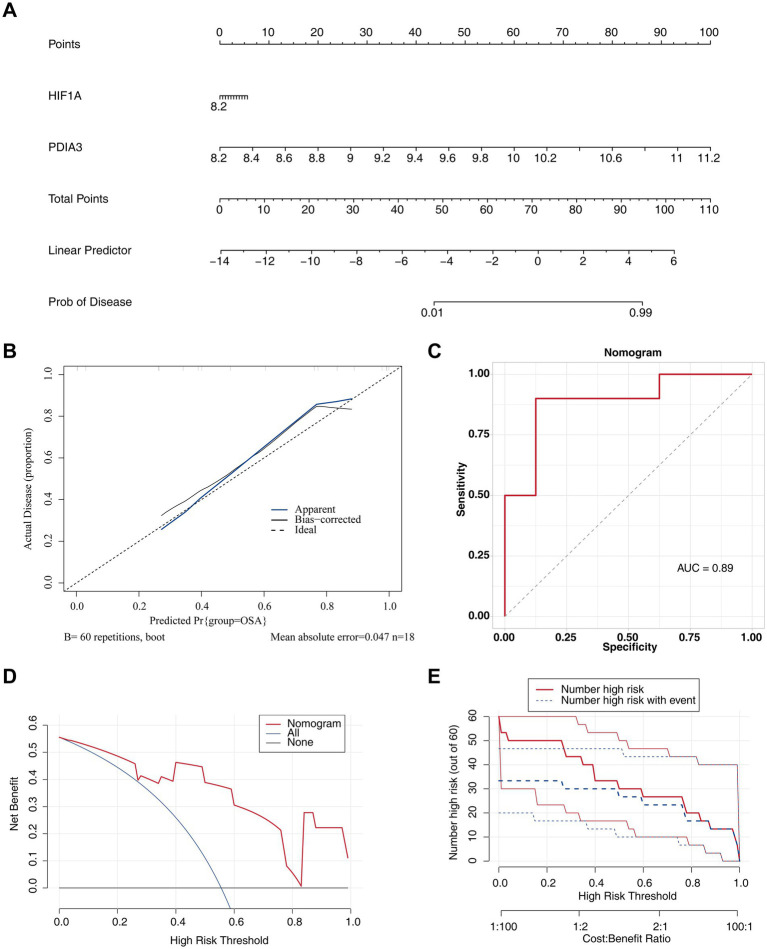
Nomogram with excellent predictive ability for OSA. **(A)** Construction of a nomogram for OSA prediction. **(B)** Calibration curve for the nomogram, with MAE = 0.047. The *x*-axis shows the predicted probability, and the *y*-axis shows the actual probability of OSA. **(C)** ROC curve for the nomogram model. **(D)** DCA for the nomogram model. **(E)** CIC showing the prediction effect of the nomogram model.

A nomogram based on *HIF1A* and *PDIA3* was developed (Figure 6A). Extensive validation of the nomogram model demonstrated robust predictive performance. The calibration curve exhibited a minimal MAE of 0.047, indicating excellent concordance between predicted and observed outcomes (Figure 6B). The ROC curve showed an AUC of 0.89, highlighting the model’s strong discriminatory capability (Figure 6C). Decision curve analysis confirmed clinical utility, with net benefits exceeding zero (Figure 6D). Moreover, in the CIC, the “Number high risk” curve consistently outperformed the “Number high risk with event” curve (Figure 6E).

### 3.5 Recognition of biomarker enrichment pathways

**Figure 7:**
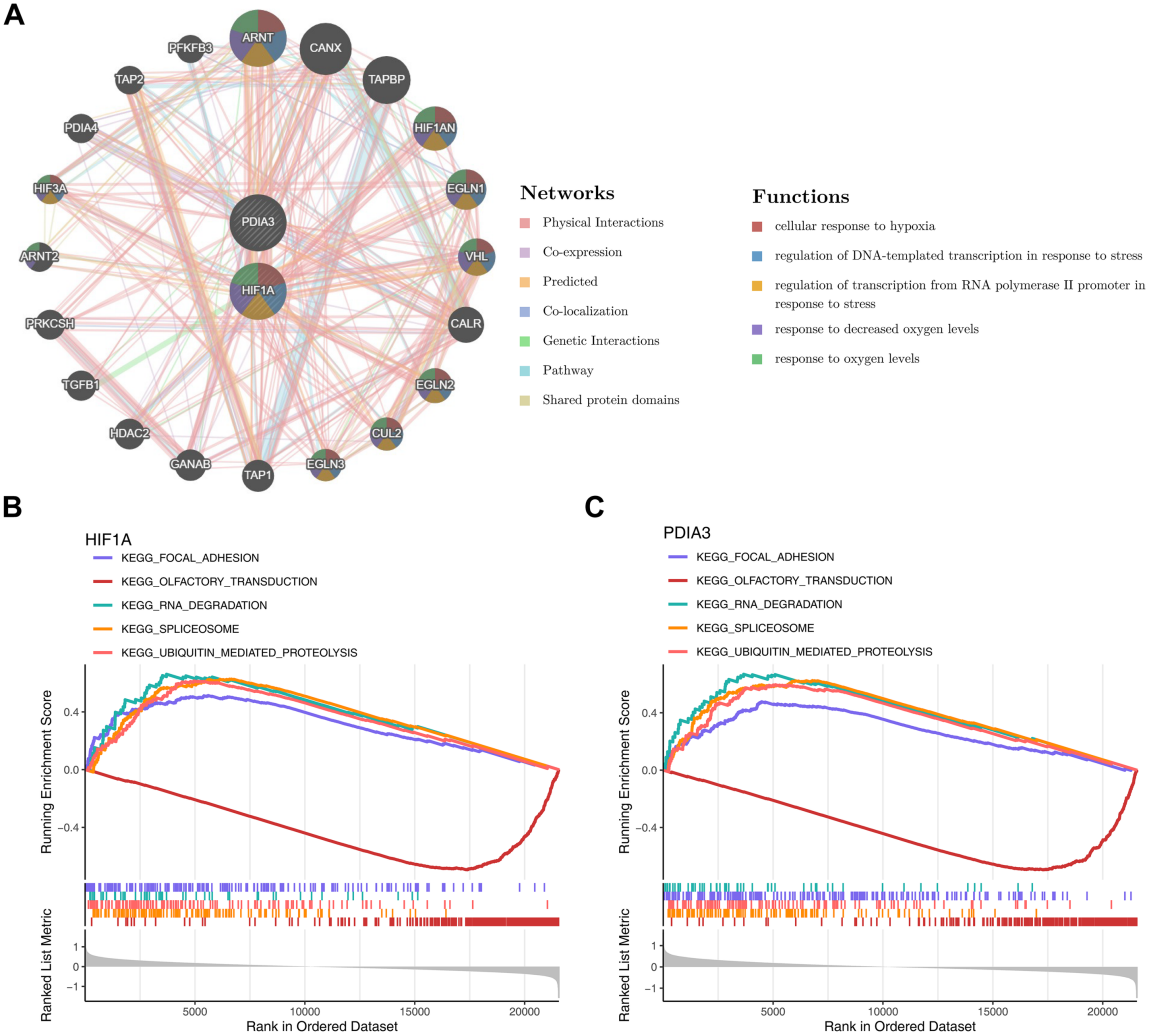
Biomarker enrichment pathways. **(A)** GeneMANIA network analysis of biomarkers. **(B)** GSEA analysis of hub gene: KEGG pathways enriched by HIF1A. **(C)** GSEA analysis of hub gene: KEGG pathways enriched by PDIA3.

Genes associated with the function of the biomarkers included *ARNT*, *HIF1AN*, *VHL*, *CUL2*, and others, with common roles in processes such as “cellular response to hypoxia,” “regulation of oxygen levels,” and “adaptation to decreased oxygen levels” (Figure 7A). GSEA indicated that *HIF1A* and *PDIA3* were co-enriched in pathways like “focal adhesion,” “olfactory transduction,” “RNA degradation,” “spliceosome,” and “ubiquitin-mediated proteolysis” (Figures 7B,C).

### 3.6 Biomarkers were modulated by multiple factors and drugs

**Figure 8:**
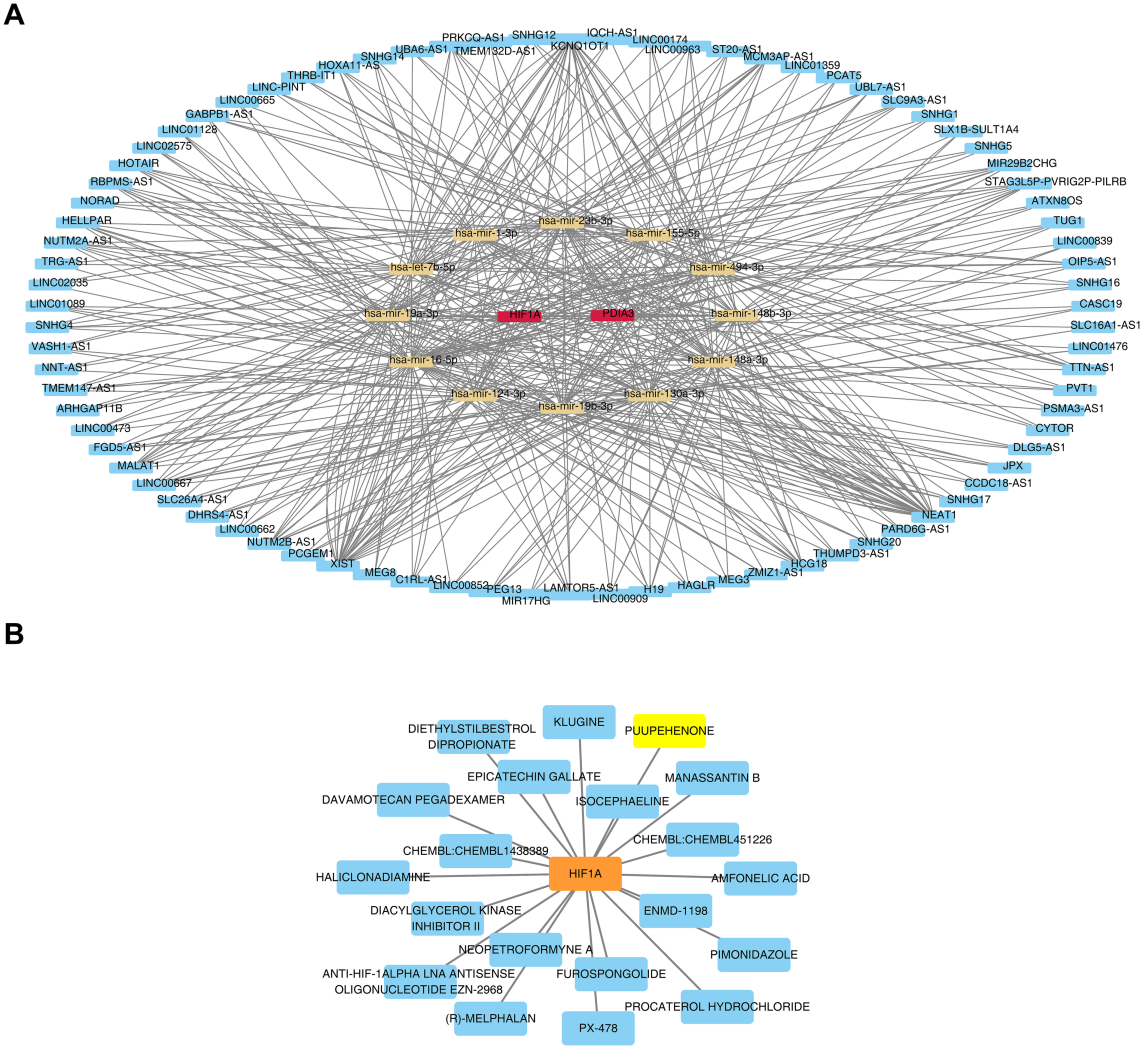
Comprehensive regulatory network analysis: lncRNA-miRNA-biomarker interactions and biomarker-targeted drug network **(A)** lncRNA-miRNA-biomarker regulatory network: red represents the genes, yellow represents the miRNAs, and blue represents the lncRNAs. **(B)** Construction of a biomarker (HIF1A)-drug interaction network.

Using the miRNet database, 220 miRNAs targeting the biomarkers were predicted, including a core subset of 15 miRNAs that simultaneously targeted both *HIF1A* and *PDIA3*. From these 15 core miRNAs, a broader network of 316 lncRNAs was identified. A lncRNA-miRNA-mRNA regulatory network was constructed with a degree threshold of ≥3, encompassing 2 biomarkers, 12 miRNAs, and 82 lncRNAs. This network revealed various regulatory relationships, such as CYTOR-hsa-miR-1-3p-HIF1A, CYTOR-hsa-miR-1-3p-PDIA3, NEAT1-hsa-miR-124-3p-HIF1A, and NEAT1-hsa-miR-124-3p-PDIA3 (Figure 8A and Supplementary Table 2). In drug prediction analysis, only drugs targeting HIF1A were identified, including klugine, puupehenone, and isocephaeline (Figure 8B).

## 4 Discussion

OSA, a condition characterized by a range of pathological and physiological changes resulting from chronic hypoxia, increases the risk of cardiovascular diseases, diabetes, and malignancies (34). Despite its significant health impact, OSA diagnosis remains primarily clinical, relying heavily on nocturnal polysomnography (PSG), which limits diagnostic yield. Protein palmitoylation, a critical post-translational modification involved in numerous diseases, has been underexplored in the context of OSA (35). This study employed bioinformatics approaches to investigate the biological pathways and regulatory mechanisms involving PRGs, with a focus on *HIF1A* and *PDIA3* as biomarkers, providing new avenues for OSA research and treatment.

Hypoxia-inducible factor 1-alpha (*HIF1A*) plays a pivotal role in mediating the transcriptional response to hypoxia, serving as a major regulator of the hypoxic response. HIF1A is also implicated in tumor immunity (36), angiogenesis, metabolic processes, and cell cycle regulation (37). *HIF1A* is recognized as a biomarker linked to cancer aggressiveness in OSA (38). *HIF1A* is a major regulator of oxygen metabolism homeostasis, while OSA is characterized by IH, in which case *HIF1A* may be activated, regulating the adaptive response of cells to hypoxia, which in turn affects the pathophysiological process of OSA (39, 40). Chronic IH, a hallmark of OSA, induces low-grade systemic inflammation, which in turn elevates *HIF1A* expression in patients with OSA (1, 42, 43). Elevated levels of *HIF1A* in the plasma of patients with OSA have been reported (44), corroborating the findings of this study. The consistent upregulation of *HIF1A* further supports its potential as a diagnostic biomarker for OSA. Although *HIF1A* is a common response to hypoxic environments, its non-specificity may affect its efficacy as a solitary biomarker. However, through further research on its variations in the early stages of OSA, its potential for early diagnosis of the disease may be discovered. Protein disulfide isomerase family A member 3 (*PDIA3*), a chaperone within the PDI family, is highly expressed in response to cellular stress and helps prevent apoptotic cell death associated with endoplasmic reticulum (ER) stress and protein misfolding. *PDIA3* has emerged as a diagnostic marker for OSA (45). IH caused by OSA can lead to ER stress (47). *PDIA3*, as a molecular chaperone in the endoplasmic reticulum, facilitates the correct folding of proteins and alleviates ER stress (48, 49). By regulating the ER environment, *PDIA3* may mitigate cellular dysfunction and metabolic impairment induced by OSA. And *PDIA3* is frequently overexpressed in various tumors, serving as a potent pan-cancer prognostic biomarker (50). A meta-analysis has suggested that OSA increases cancer risk (51). This indicates that *PDIA3* may serve as part of a set of biomarkers, providing new insights for clinical diagnostic research of OSA. In clinical applications, the combined detection of *HIF1A* and *PDIA3* expression levels in blood or saliva samples may enhance the diagnostic capability for OSA: an increase in HIF1A may indicate acute hypoxic events, while sustained high expression of PDIA3 may reflect chronic pathological damage. Furthermore, alongside clinical symptoms such as nocturnal apnea and daytime sleepiness, this combined biomarker strategy is expected to simplify the stratified diagnosis of OSA. In the future, it is necessary to verify its sensitivity and specificity through prospective cohorts, explore its synergistic effect with other markers (such as inflammatory factors), design a joint diagnostic model, and develop a multi-biomarker combined detection strategy to optimize the individualized diagnosis and treatment pathway of OSA.

Based on GSEA results, *HIF1A* and *PDIA3* shared five common enrichment pathways, including focal adhesion, olfactory transduction, RNA degradation, spliceosome, and ubiquitin-mediated proteolysis. In OSA, IH activates *HIF1A*, which in turn triggers pro-oxidase genes, leading to the production of reactive oxygen species (ROS) within cells (52). Excessive ROS can induce oxidative stress, which may alter cell morphology and function, including the formation of intracellular punctate focal adhesions (53). This finding aligns with the results of this study, which confirmed the up-regulation of *HIF1A* and *PDIA3* in the focal adhesion pathway. OSA-related biomarkers are significantly enriched in olfactory pathways (55). In this study, olfactory transduction was predominantly associated with down-regulated genes, consistent with earlier findings (54, 56). However, the specific mechanisms linking the focal adhesion and olfactory pathways to OSA require further investigation. The ubiquitin-mediated proteolytic pathway has been linked to various diseases, including malignancies (14, 57), Parkinson’s disease (41), chronic obstructive pulmonary disease (46), insomnia (58), and arthritis (41), but its association with OSA has not been explored. This study is the first to identify a relationship between OSA and the “RNA degradation” and “spliceosome” pathways, with the underlying mechanisms warranting further research.

miRNAs are small, non-coding RNA molecules that regulate post-transcriptional gene expression and influence a variety of physiological processes. The downregulation of miR-124-3p promotes cancer growth and metastasis across different tumor types (59), and the incidence of tumors is higher in patients with OSA (60). Additionally, miR-124-3p is up-regulated in the IH environment (59), suggesting that it may play a protective role in tumorigenesis and progression in OSA individuals with cancer. Furthermore, the upregulation of miR-1-3p inhibits solid tumor growth in various tissues, interacting with β-catenin (61). By inactivating the Wnt/β-catenin pathway, miR-1-3p may contribute to cognitive impairment in patients with OSA (62). The expression of miR-23b is elevated in patients with OSA and correlates positively with disease severity. HIF-1 is involved in the upregulation of miR-23b, which, in turn, regulates the feedback loop of HIF-1 expression (38). This illustrates the role of miRNAs in the progression of OSA and its association with tumor development. LncRNAs are large RNA transcripts that do not encode proteins but can mediate gene expression by interacting with DNA or chromatin regulators in the nucleus (63). Metastasis-associated lung adenocarcinoma transcript 1 (MALAT1) and nuclear paraspeckle assembly transcript 1 (NEAT1) are both upregulated in the tissues of patients with OSA and play roles in apoptosis, inflammation, and oxidative stress induced by IH (64). NEAT1 aggravates endothelial cell injury in individuals exposed to IH through the Apelin/Nrf2/HO-1 signaling pathway (63), potentially exacerbating OSA. These findings suggest that lncRNAs are involved in the development of OSA.

This study also explored potential therapeutic agents for OSA. Antioxidants have been recognized as promising treatments for OSA (65). Puupehenone, a sesquiterpene quinone isolated from sponges, demonstrates strong antioxidant properties (66) and can inhibit human lipoxygenase (LOX). LOX promotes the production of ROS (67), which are significantly elevated in patients with OSA. LOX is also involved in the synthesis of leukotrienes from arachidonic acid, a critical step in the inflammatory process (68). Thus, puupehenone’s antioxidant properties may help mitigate the inflammation induced by OSA-related hypoxia. Klugine and isocephaeline inhibit hypoxia-induced HIF-1 activation by blocking the accumulation of HIF-1α protein, which is highly expressed in patients with OSA (69). These two drugs may offer potential therapies for OSA. PX-478, an orally active HIF-1α inhibitor, has shown significant therapeutic effects in patients with OSA and tumors (60), suggesting its potential for future pharmacological treatment of OSA.

However, this study also has some limitations. Firstly, the sample size in the dataset obtained from public databases is limited, and the information about the samples is not sufficiently detailed, which may affect the accuracy and generalizability of the results. Secondly, the specific mechanisms of action and diagnostic value of *HIF1A* and *PDIA3* in OSA require further experimental verification. Therefore, we plan to collaborate in the future to collect a larger scale of OSA patients and healthy control samples, while thoroughly documenting the clinical characteristics of patients (such as disease duration, severity, comorbidities, etc.) to enhance the representativeness of the data and the reliability of the results. At the same time, we will conduct cell and animal experiments to establish an OSA disease model, deeply explore the molecular regulatory pathways of *HIF1A* and *PDIA3* in the occurrence and development of OSA, clarify their mechanisms of action through gene knockout and overexpression techniques, and integrate findings from a large clinical cohort to validate the efficacy of both as diagnostic markers, thereby providing a more solid basis for the diagnosis and treatment of OSA.

## 5 Conclusion

In this study, bioinformatics analysis identified two biomarkers associated with palmitoylation in OSA: *HIF1A* and *PDIA3*, which can serve as diagnostic biomarkers and therapeutic targets for OSA. Additionally, several potential targeted drugs were identified, which may have significant therapeutic effects on OSA. These findings provide new insights and directions for OSA treatment.

## Data Availability

Publicly available datasets were analyzed in this study. This data can be found here: http://www.ncbi.nlm.nih.gov/geo/.

## Footnotes

^1^ http://www.ncbi.nlm.nih.gov/geo/

^2^ https://string-db.org/

^3^ https://rdrr.io/github/yikeshu0611/ggDCA/

^4^ https://genemania.org/

^5^ https://www.mirnet.ca/

^6^ www.dgidb.org

## Glossary

OSA: Obstructive sleep apnea
PRGs: Palmitoylation related genes
DE-PRGs: Differentially expressed PRGs
IH: Intermittent hypoxia
ZDHHC: The zinc finger protein DHHC
GEO: The Gene Expression Omnibus
FC: Foldchange
ssGSEA: The single-sample gene set enrichment analysis
MM: Module membership
GS: Gene significance
GO: Gene Ontology
KEGG: Kyoto Encyclopedia of Genes and Genomes
BP: Biological process
MF: Molecular function
CC: Cellular component
STRING: The Search Tool for the Retrieval of Interacting Genes
PPI: Protein–protein interaction
MCC: The maximum cluster centrality
LASSO: Least absolute shrinkage and selection operator
SVM-RFE: The Support Vector Machine-Recursive Feature Elimination
ROC: Receiver operating characteristic
AUC: Area under the curve
MAE: A mean absolute error
CIC: Clinical impact curves
MSigDB: Molecular Signatures Database
FDR: False discovery rate
GSEA: Gene set enrichment analysis
NES: Normalized enrichment scores
miRNAs: microRNAs
lncRNAs: Long non-coding RNAs
DGIdb: Drug-gene interaction database
HIF1A: Hypoxia inducible factor 1 alpha
PDIA3: Protein disulfide isomerase family A member 3
ER: Endoplasmic reticulum
ROS: Reactive oxygen species
MALAT1: Metastasis-associated lung adenocarcinoma transcript 1
NEAT1: Nuclear paraspeckle assembly transcript 1
LOX: Lipoxygenase
