# Supplementary material for: Identification and molecular mechanism of palmitoylation-related biomarkers in obstructive sleep apnea
Source: Front Neurol. 2025 Aug 29;16:1499573. doi: 10.3389/fneur.2025.1499573 (PMC12426112; doi:10.3389/fneur.2025.1499573)
Supplement: Supplementary file 5 [file Table_3.docx]

Supplementary Material

# Supplementary Data

**Supplement Table 1** Sample information of GSE135917.

**Supplement Table 2** Predictions for lncRNA and miRNA.

**Supplement Figure 1** Protein-protein interaction (PPI) network constructed based on candidate genes.

**Supplement Figure 2** Expression levels and ROC analysis of LUM and TXN. (A-B) Box plots of the expression levels of LUM and TXN in the GSE38792 and GSE135917 datasets. ns represents P > 0.05, * represents P < 0.05, and ** represents P < 0.01. (C-D) ROC curves of LUM and TXN in the GSE38792 and GSE135917 datasets.
